# How the world of biobanking is changing with artificial intelligence

**DOI:** 10.3389/fdgth.2025.1626833

**Published:** 2025-09-03

**Authors:** Michaela Th. Mayrhofer

**Affiliations:** ^1^ Institute of Human Genetics, Medical University of Innsbruck, Innsbruck, Austria; ^2^ Papillon Pathways e.U., Landskron, Austria

**Keywords:** biobank, artificial intelligence, datafication, health economy, size, site, access, speed

## Abstract

Artificial Intelligence is increasingly shaping the practice of biobanking by influencing how biobanks evolve and operate, especially when it concerns their relationship to data. By assessing four key parameters—size, site, speed, and access—this paper analyzes the impact of AI technologies on biobanks, presenting them as dynamic boundary objects that produce biovalue by transforming biological material and data into intangible assets of the data-driven bioeconomy. Historically rooted at the intersection of health research and healthcare, biobanking is continually reshaped by emerging technologies, policies, and societal expectations. While biobanks were originally defined as collections of samples and associated data, they have recently evolved into complex infrastructures for both data and samples.

## 1 Introduction

Biobanking is here. Artificial Intelligence (AI)-driven process automation, data analytics, robotics, the internet, and other rapidly emerging technological advances are driving the revolution of biobanks, biorepositories, and biospecimen science. With the evolution of biobanking from a simple collection of frozen specimens to the virtual biobanks and bioscience seen today, the rise of biobanks brings each nation and its healthcare and economic systems a transformative potential. (1).

The cited passage originates from the exposé titled “Biobanking is Changing the World”, which was published in *Forbes Magazine* about half a decade ago. While many facets of biobanking have been studied extensively throughout the years by various academic disciplines (and continue to be examined), *Forbes Magazine* looked at biobanks with an entrepreneurial lens. Generally, *Forbes Magazine* features trade news and financial information aimed at a target readership in business and technology. As regard to the above excerpt, it is therefore worth noticing that much emphasis is placed on biobanks' “transformative potential” that is—when unlocked by AI—driving both societal progress and economic prosperity (e.g., “human health” or “nation's economy”), presenting this as a novelty to the business world.

Rather than being something ‘new’ in and of themselves, biobanks can look back as organized sites that support and satisfy medical curiosity since the 16th century (2). Since a couple of years, biobanks are broadly defined as collections of biological samples and associated data (3), typically located at the intersectoral space between healthcare and health research. Since the late 1990s, the practice of biobanking has gained new meaning—especially for the life sciences—due to standardization, professionalization and the assemblage of critical mass in both material resources and expertise (4). Consider, moreover, the ISO 20387:2018 definition of biobank as a “legal entity or part of a legal entity that performs biobanking” and biobanking as the “process of acquisitioning […] and storing, together with some or all of the activities related to collection, preparation, preservation, testing, analyzing and distributing defined biological material as well as related information and data.”^1^ Subsequently, “biobanker” became a distinguished career path that is accredited through post graduate training courses in overall management, data quality, or regulatory and quality aspects.^2^ Today, biobanks can exist either as stand-alone entities^3^ or integral parts of a clinical infrastructure, especially within university hospitals.^4^ They may be part of transnational networks or research infrastructures (5) and are often considered valuable for private-public partnerships (6), translational research (7) or identified as symbolic locations of national identity (8) or as sites that generate and express bioeconomy (9), and support the interpretation that samples are not isolated objects but also function as data in themselves due to their embedded informational content that is derived through (biomedical) analysis (10).

The *Forbes Magazine* article is now approximately five years old and seems to have aged well. It is thus a good time to revisit its key arguments, especially in relation to the effects that are brought to light through the progression and implementation of AI technologies. As a starting point, it can be noted that countries continue to set up or maintain national biobanks [e.g. (11)]. Second, new technologies such as AI not only shape innovation, but a data-driven world has led to a global race of nations for AI dominance (12). Third, several countries strive to strike for a balance between consumer/citizen/patient rights on the one hand, while enabling open access to data infrastructures and combine data sets to stimulate entrepreneurial innovation on the other hand [e.g. (13)].

The three observations listed above are not comprehensive. Rather, they allow for a suitable argumentative opening to investigate how specific factors have shaped the intersectoral space between healthcare and health research. AI is one of many technologies that affect and shape the practice of biobanking. AI-based algorithms, when applied to biobank data, for instance, can accurately categorize phenotypes by enabling metabolite mapping and demonstrate future clinical applications (14). Alternatively, efficiency is increased by speeding up the analyzation and labeling of images in shorter timeframes by training AI-tools on imaging data from biobanks (15). However, transformations like these, are not solely driven by technological advancements. They emerge in a regulatory environment and ethical framework that guide their implementation. As such, AI technologies situated in the practice of biobanking serve as a crucial lens through which we can explore the evolving landscape of health data governance, data privacy concerns, and the shifting dynamics between technological innovation and public policy. Thus, following, this brief introduction, the next section of this article discusses the ever contingent and changing practices of biobanks by examining the parameters of size, site, access and speed in relation to AI's potentiality for biobanking.

## 2 Artificial intelligence: impacting size, site, access and speed in the data-driven health economy

While the collection and categorization of biological materials has developed into an organized practice since the 18th century, computerized databases have supplemented the practice of sample processing and have—since the 1980s—been steadily integrated into the laboratory and scientific work of the biological and biomedical sciences (16, 17). This progression prompted Timothy Lenoir (18) to note that databases challenge laboratories as primary sites of knowledge production, and with it anticipated what Anne Beaulieu called the “informational turn” (19), which describes the turn to data as the now dominant source for scientific knowledge production. Subsequently, data was depicted as a critical resource, exemplarily portraying big data as the “oil of the information economy” (20) or describing national population registers as “goldmines” (21).

Data intense practices share—as noted by many—that they have an unquenchable thirst for ever more data (20, 22). At the same time, especially the health sector is driven by the conviction that datafication will lead to open innovation and precision medicine, including economic growth (23). Snell and others have formulated this development as the turn towards a “regime of data-driven health economy” (24). This regime does not only run on the insatiable thirst for big data, but also on the promise of infinitely commercially exploitable possibilities. Perhaps most crucially, the authors identified the following paradox: namely, that today's system of a data-driven health economy is constructed on the data collection mechanisms of the welfare state, which builds on both the principle of solidarity and a functioning social contract. However, when data extraction is redirected toward private profit and contributes to the erosion of public (health care) systems, the legitimacy of such data practices is called into question. As they argue, “[t]his contradicts the justification of the welfare state data gathering, since a promise of profit itself is not necessarily enough to justify the uses of citizens' personal data as a resource for economic activity and wealth outside data's original, primary context.” (ibid).

Health data today is gathered for many purposes, among them personalized medicine, scientific discovery, disease prevention, lifestyle or self-management of care. The health data industry is a growing sector, and global companies such as Google or Amazon are expanding their ventures into health research and health care (25–27). The employment of health data promises early detection, increased health literacy or tailored therapy. On that ground, the effective utilization of data has become a central objective of health and innovation policies worldwide, including reform plans for national healthcare systems. National strategies seek to harness the potential of health data management in order to enhance (cost- and time-) efficiency in patient care, to enable the stratification of personalized medicine, and to foster research and development—while simultaneously negotiating concerns related to efficiency, fairness, (bio)value, and potential exploitation or discrimination, among other issues (28–31). Moreover, many scholars, while arguing for the employment of AI technologies, do so with caution and call for the study of AI and big data associated risks such as data biases or exclusion criteria (15, 32, 33), especially because data is seen as all-powerful but not as innocent—or as Kelly Bronson described it, “immaculately conceived” (34).

AI algorithms—powered by (big) data—are one of many tools for enabling transformative innovations that influence and shape the practice of biobanking. Due to its perceived “potentiality”, AI has thus become one of the most widely discussed and both morally and economically invested technologies in recent years (71).

In the following sub-chapters, we will employ an AI lens to assess how the practice of biobanking is formed by several key parameters. For this paper, these are size, site, access, and speed. These four parameters serve as the foundation for constructing an analytical framework through which to observe the continuous evolution of a biobank, biobank network or even infrastructure by mobilizing the analytical tools of “boundary objects” (35), “biovalue” (36) and “assetization” (37). Assetization describes how assets are created through both tangible and intangible valuation practices that assign value to resources or concepts and transform them into economic assets. Biovalue refers to the value produced by the biotechnological reformulation of living processes into something else, whereas boundary objects can be defined as entities that operate at the intersection of multiple disciplines and facilitate the meaningful translation of practices across them. Employing these three concepts within the parameters of size, site, access, and speed, ultimately permits describing how biobanks operate at the crossroad between health care and health research by transforming data, samples and new technologies such as AI into assets and therewith generating value for the bioeconomy.

### 2.1 Size

Let us firstly assess the significance of size by highlighting its importance as a statistical requirement. This is particularly evident in epidemiology, where large datasets are a precondition for any meaningful statistical analysis. Equally so, new research findings necessitate to re-assemble even bigger quantities of fit-for purpose or high-quality datasets. Consequently, quality management standards must be defined, implemented and checked to ensure, for example, reliability in sample and data analysis, compliance with ethical and legal requirements for (re)use, or clarity regarding data provenance (38, 39). If quality standards are not met, the principle of “garbage-in, garbage-out” (40) would reduce the utility of a biobank not only by volume but by lack of scientific value. In the worst case, a biobank would no longer be considered an asset in the bioeconomic sense (37), and thus without any value at all.

In relation to AI, it is suggested that some well-designed algorithms only need a small, but high-quality dataset to be appropriately trained for purpose, unless it concerns deep learning algorithms that require big data:

The size of the dataset required is directly proportional to the type of AI used and its field of application. Even a large dataset may not be useful if it is noisy, incomplete, or biased. A primary issue is the problem of complex, highly specialized, and specific fields focusing on molecular interactions, protein structures, or drug discovery that typically require domain expertise and specialized knowledge. As a result, the problem space is more constrained, and the available data may be more targeted and focused. In such cases, a smaller sample size can still provide meaningful insights and accurate predictions. (14).

Put differently, data and sample quality are integral aspects of defining the size of what constitutes a “critical mass”. This again requires collaboration practices, which rare disease biobanks were the first to understand:

Another challenge is that RD biobanks need to be connected within networks that ensure uniformly high quality levels of both biomaterials and associated clinical data, apply harmonized operational procedures, reduce redundancies, optimize investments, and facilitate exchanges of expertise and competences. (41).

We have thus established that size matters, but to which degree and why is context dependent and not exclusively linked to statistical power. Paul Burton and others argued that “from a strategic perspective, it is still unclear what ‘large enough’ really means. This question has critical implications for governments, funding agencies, bioscientists and the tax-paying public. Difficult strategic decisions with imposing price tags and important opportunity costs must be taken” (42). Consequently, aspects of size are used for reporting success stories and justifying investments in infrastructure (incl. community) building: “The following biobanks are some of the largest in the world […]”^5^ or “EBW25 [congress] for the biggest biobank networking opportunity”.^6^ In other words, clinical and national biobanks have detected that beyond the statistical power of scale, there is also the epistemic power of size that can be employed to funders, customers, or biobankers for creating a sense of pride and purpose. This is equally true when describing the biobank as a national asset and evoking national pride:

When biobanks furthermore acquire a size facilitating claims about national representativity, they potentially come to embody the nation in an almost somatic sense. Biobank freezers can be used metaphorically as a proxy for the surrounding society (I remember once a biobank representative told me that the freezers were where they “kept 80,000 people”), and therefore it is no surprise that large-scale biobanking can become arenas for public negotiation of the duties, entitlements, and mutual obligations between state and citizen. (43).

Ultimately, size goes beyond the quantitative, as Klaus Hoeyer eloquently argues. While size may initially appear to be a neutral, objective, and purely quantitative measure, it is far from an apolitical category. Rather, size often carries embedded assumptions and expectations, strategic implications as well as power dynamics. Consequently, the category of size can be leveraged not only to establish statistical relevance or provide evidence-based justification for a scientific argument, but also to promote a particular collection or biobank strategically, thereby promoting and trading the biobank's assets, justifying sampling and access strategies or legitimizing the allocation of public funds.

### 2.2 Site

Let us now consider the category of site which can be physical or virtual, centralized or federated, legalized or a social assemblage. Science and technology studies conceptualize assemblages as relational, experimental, and situated in the context of institutionalized multidisciplinary and/or transnational science collaboration. Assemblages emerge, when certain elements—whether they are material and immaterial in their nature—come together and collectively stabilize a social system such as when a biobank or infrastructure is implemented through discourses, practices, technologies or norms in a specific moment (44). In this sense, biobanks can be described as sites where the materiality of both samples and data is transformed into tangible assets with biovalue, which play a pivotal role for the bioeconomy.

Human genomics is undergoing a step change from being a predominantly research-driven activity to one driven through health care as many countries in Europe now have nascent precision medicine programmes. To maximize the value of the genomic data generated, these data will need to be shared between institutions and across countries. In recognition of this challenge, 21 European countries recently signed a declaration to transnationally share data on at least 1 million human genomes by 2022. (45).

For expanding on this quote further, the “living organism” metaphor proves useful as it helps depicting the temporal dimension of and spatial organization. It indicates that a biobank, network or infrastructure does not miraculously appear, but is “assembled”, constantly evolving. Just as for an organism, the evolution of a biobank corresponds to the interplay of maturity and social dynamics (46). Biobanks or research infrastructures, in and of themselves—regardless of the sector in which they are implemented—are more than technical constructs. Rather, as Melissa Gilbert and others (47) have argued, they are social systems that play a key role in shaping societal transformations. This is particularly evident when it concerns aspects of equity, inclusion and justice, where the design and conventionalization of infrastructures can either reinforce or obliterate existing disparities. Building on the arguments laid out by Susan Leigh Star and Karen Ruhleder (48), these assemblages of technological and social knowledge might assemble and translate as infrastructures that may be tangible/intangible, federated/centralized, product/process or material/immaterial. Moreover, a village is required, so Christine L. Borgman and Paul Groth (49), to overcome the physical and social distances that lie between data creators, data reusers, data curators or funding agencies. Their understanding goes beyond mere situatedness in a particular context. Rather, they argue that the relational aspects affect the social and technical distances through time and temporality.

We can take from this, that it requires a constant dialogue between the stakeholders to find means to overcome the spatial gap, which is complex and requires infrastructure. “Boundary object,” a concept used in science and technology studies to describe entities that lie at the intersection between communities, is helpful here to localize the space biobanks shape (35). At the same time, infrastructures become more and more complex while striving to sort things out through classification techniques (50). In relation to AI, known aspects such as especially trustworthiness (e.g., human decision making) and economic exploitation (e.g., intellectual property rights or data sovereignty), become more prevalent.

### 2.3 Access

Let us now turn to the category of access, which is a critical one for any biobank or any data infrastructure as they do not have much value if they cannot be accessed. To manage accessability, access committees typically define, implement, and monitor the conditions of use, which are a key part of any governance framework and strategy. Consider, for instance the European Union's ambitious European Health Data Space (EHDS) initiative, which aims to grant citizens increased access to and control of their (electronic) health data across the EU, whilst facilitating health data re-use for public and private research and innovation, as well as policymaking. This initiative clearly positions health data as an asset for primary care but also for research and innovation by defining as one of its key goals to generate public value by increasing access to health data by balancing simplified access for Big Tech innovation without risking the solidarity-based health care systems. Put differently, “the aim of stimulating the European economy by granting free access to citizens' health data can backfire and have detrimental effects on public trust in and support for medical research” (51). If done wrongly, it can even reinforce digital divides and social inequalities (52). This positions the envisioned national health data access bodies (HDABs) as critical gatekeepers—and possibly bottlenecks—in the implementation of the EHDS. To date, however, HDABs lack a clear and practical mandate, especially because the secondary use of health data derived from electronic health care records is not specifically defined in Article 34.^7^ In addition, access conditions to health data is difficult to harmonize across Europe due to a plethora of diagnostic codes or standards that challenge interoperability:

Even if all EU countries should begin using ICD-11, the same diagnostic codes will be used differently and signify different stages of disease in healthcare systems with different remuneration systems, varying access to healthcare, and diverse registration traditions. Arriving at agreements on semantics and data management procedures among many different stakeholders is a monumental challenge. Indeed, implementation of new EHR [electronic health care record] systems is very time-consuming, costly, and largely beyond healthcare professionals' remit and scope of action. It is also very challenging, even within a limited (disease or national) area, let alone the entire Union. (51).

This challenge for data sharing across countries is well described in scientific literature, among which the FAIR principles, which stand for “findable, accessible, interoperable, and reusable” (53), are the most prominent ones. Intended as guiding data policy instrument for improved data management across all sciences, the FAIR principles are not only widely accepted in the research community but also heavily promoted by policy makers hoping to stimulate a greater (re)use of health data. To implement FAIR for the practice of biobanking, biological material and data are best conceived as a unified resource. It enables the integration of comprehensive provenance information—including data reuse. It does, however, not provide any indication about fair access. This is not to diminish the importance of technical principles such as FAIR, but rather to emphasize that they are not sufficient on their own to deliver on the complex governance and sensitive data management that is needed to preserve public trust. Enabling fairness requires extensions of the very same principles by additionally promoting data quality, incentivizing data sharing and upholding ethical and privacy preserving practices as suggested by FAIR-health (54). Alternatively, FAIR-er (55) promotes the inclusion of engagement and participation mechanisms in the design of data governance frameworks. While a lot has been achieved, a high level of complexity remains to make access both FAIR and fair, even in the most advanced countries in health data digitalization, let alone across Europe for the realization of the EHDS (68, 69). Consider, in this context, the example of Findata,^8^ that is designed to grant permits for the secondary use of social and health care data while improving data protection for individuals as a one-stop-shop therewith simplifying access conditions (24).

In the broader context of biobanks and data repositories, the use of AI on health data undoubtedly highlights the interrelated aspects of technology, standards and fair regulation even further. It creates tension between the promotion of AI technologies and data privacy concerns, illustrating how data hunger conflicts with the principle of data minimization (56). Through the deployment of AI, the intertwined aspects of ethical compliance and technical standards become more visible and often require ethical trade-offs inherent to data-intensive practices. “Countries must thus decide how to balance the positive goals of secondary-use activities like healthcare AI with mitigating associated privacy risks. These trade-offs raise issues of resource allocation and justice that have so far been largely neglected in policy debates and the scholarly literature” (57). This especially culminates when access conditions are defined and negotiated, and the authors call for a broader ethical debate on funding priorities rather than just holding AI systems accountable. “This of course requires transparent insight into the available budgets and competing needs. All in all, if such reflections lead to a country explicitly deciding to focus on a strict, conditional or liberal approach to data privacy and/or data access, that decision is morally legitimate if it fulfils conditions of procedural fairness, e.g., accountability and transparency” (57).

Consequently, as many scholars have pointed out, the integration of AI technologies in the healthcare and health research sector must strike a careful balance between free-market forces and open access policies that align with fair (small caps) access, especially when supporting a solidarity-based health system rather than undermining it. For addressing this challenge, digital health strategies need to be developed that are both coherent and in line with the fundamental constitutional values such as rule of law or human dignity. Including such values in the overall data governance and access policy frameworks is a key ingredient for nurturing trustworthiness (24, 57–59). Accordingly, any “boundary object”—such as biobanks situated at the intersection of health care and health research, or AI technologies, increasingly embedded across all sectors of contemporary life—must be aligned with both technical and ethical data access frameworks, integrated within robust governance structures, and, perhaps most importantly—coherently incorporated into the respective health(care) system. Failing to do so risks rendering citizens into commodified objects of an asymmetrical bioeconomy rather than empowered subjects.

### 2.4 Speed

It is uncontested that innovation in AI is emerging at an exuberant speed. In the world of business and innovation, speed is a category of its own—especially, when it provides a head start and advantages over competitors. While we cannot do full justice on this aspect, it is critical to point out that one, an AI race for global dominance is in full swing and two, it is widely supported by ambitious national strategies and substantial public and private investments (60, 61). Some are in this race to win, whereas others entered the race not to be left behind. In general, as Holzinger and others remind us, “digital transformation can involve the introduction of new technologies and processes to improve the efficiency, accuracy, and speed of research and development and enable the development of entirely new and disruptive products and services” (62).

When looking at the category speed in relation to the integration of AI tools into health research (that embraces innovation by default) and healthcare (that is cautious to change by default), the category of speed is predominantly linked to narratives of efficiency and promises of enhancement of the productivity of research processes and patient care.

Especially for repetitive administrative processes or medical images, AI-assisted tools are expected to expediate, for example, diagnostic workflows by automating repetitive tasks or medical image analysis through faster pattern recognition (70). For biobanking, AI is attributed potentiality (71). It is argued that AI will transform the practice of biobanking even more to the digital space, especially in cancer research, where large datasets available in biobanks are used by machine learning applications that advance the understanding in cancer biology—while building on the decades-long know-how and efficiency of biobanks in sensitive data management (63). At the same time, there is large agreement that it is critical to preserve or build trust(worthiness) in AI systems by retaining accountability through human-in-the-loop or human-in-command approaches. This requires the translation of high-level recommendations into practical processes that can be adhered to regardless the fast pace of technological development and slow regulation (64–67).

## 3 Conclusion

By examining the categories of speed, site, size and access, this paper explored how AI technologies shape and transform the practice of biobanking, especially in relation to data. For decades, biobanks have been situated at the intersection between health research and healthcare. They have operated as boundary objects that transform the value of samples and data into assets for the bioeconomy. Put differently, biobanks do not merely store samples and data, they actively participate in the co-production of scientific knowledge or governance structures. They shape and are shaped by their environment. Whereas the practice of biobanking has become more standardized and institutionalized over time, it always was a practice that needed to remain adaptive to new technologies, regulations, societal priorities or national strategies. Biobanks, of late, have transformed into infrastructures that are experienced in engaging with a multitude of stakeholders from within the clinic, the private sector or patient advocacy. Whereas the so-called “data turn” has been unfolding over several decades, AI technologies have nonetheless accelerated the datafication of science and medicine in the last couple of years. Yet, although AI constitutes a significant sociotechnical shift—exemplified in how it reconfigures the parameters of access, site, size, and, above all, speed—it must be situated within the longer trajectory of biobanking as a dynamic and evolving practice.

## Footnotes

^1^The ISO standard is currently under revision and will be replaced by ISO/CD 20387, see https://www.iso.org/standard/67888.html (July 07, 2025).

^2^E.g., Université Côte d'Azur: https://univ-cotedazur.eu/msc/biobanks-complex-data-management/career-path (April 20, 2025).

^3^E.g., UK Biobank: https://www.ukbiobank.ac.uk (April 20, 2025).

^4^E.g., Biobank Graz: https://biobank.medunigraz.at/en/ (April 20, 2025).

^5^https://www.biobanking.com/10-largest-biobanks-in-the-world/ (April 29, 2025).

^6^https://www.bbmri-eric.eu/events/europe-biobank-week-2025/ (April 29, 2025).

^7^https://www.european-health-data-space.com/European_Health_Data_Space_Article_34_(Proposal_3.5.2022).html (April 4, 2025).

^8^https://findata.fi/en/ (April 4, 2025).
